# An Unusual Case of Hernia

**Published:** 1904-03-12

**Authors:** Wheelton Hind

**Affiliations:** Surgeon to the North Staffordshire Infirmary.


### AN UNUSUAL CASE OF HERNIA.

By Wheelton Hind, M.D., B.S., F.R.C.S., Surgeon to the North Staffordshire Infirmary.

A widow, aged 57, was admitted to the hospital under me on September 28, 1903, at 11 o'clock, who gave the following history:—Sixteen years ago she was operated on for strangulated femoral hernia on the right side. She left hospital with a soundly healed wound. Since that time she had recurrence twice and submitted to operations for the radical cure, but during the last three years has had a return of the hernia. On the evening before admission, whilst lifting, she felt something give way in the neighbourhood of the hernia, and found that her abdominal wall had given way and the intestines were protruding. She treated the matter as if of no moment and covered the bowel up with rags and went to bed. She had no treatment till her arrival at the hospital at 11 A.M. next day, more than 12 hours after the prolapse. I saw her at 1 o'clock, and found a little grey-haired old woman in considerable amount of pain, and somewhat collapsed, with fully three feet of intestine lying over the thigh and between the legs, covered partially with dirty rags, in places the bowel and mesentery were dry and brown, and the whole gut and mesentery stiff and much congested. A good deal of fluff was adhering to the gut. Under chloroform the gut was washed with lotion of perchloride of mercury, 1 in 2,000, and cleaned up as rapidly and as well as possible. An incision was then made in the skin which was grasping the bowel somewhat tightly, and the neck of the sac examined. The hernia was found to be protruding through the femoral ring, which was large enough to admit the hand. The bowel was reduced without much difficulty and kept in position by flat sponge cloths, while the sac was dealt with. The peritoneal sac was stripped from the skin and cut off close to Poupart's ligament. The edges were then securely sewn together with silk, an endeavour being made to get good adhesion by uniting strips ¾ inch broad. The redundant skin was then cut away and sutured. There was no possibility of suturing the tendinous margins, owing to stretching and wasting in an old subject. Convalescence was uninterrupted, the temperature never rose above normal during the first fortnight, the wound healed soundly, and patient left the hospital on December 1st wearing a special support, consisting of a pad supported by a belt, the upper portion of which encircled the body, and the lower the upper portion of the thigh.

The interesting points in the case are: (1) the rarity of spontaneous rupture of a hernial sac, which probably would not have happened if the skin had not consisted largely of scar tissue; (2) the absence of shock; (3) the absence of septic infection, although the bowel was lying exposed to all manner of septic clothing in one of the most insanitary regions of the body.

